# Development and preliminary evaluation of a communication skills training programme for hospital physicians by a specialized palliative care service: the ‘Teach to Talk’ programme

**DOI:** 10.1186/s12909-020-02275-2

**Published:** 2020-10-15

**Authors:** S. Tanzi, L. De Panfilis, M. Costantini, G. Artioli, S. Alquati, S. Di Leo

**Affiliations:** 1 Palliative Care Unit, Azienda USL- IRCCS Reggio Emilia, Reggio Emilia, Italy; 2 Clinical and Experimental Medicine PhD Programme, University of Modena and Reggio Emilia, Modena, Italy; 3 Unit of Bioethics, Azienda USL- IRCCS Reggio Emilia, Reggio Emilia, Italy; 4 Scientific Directorate, Azienda USL-IRCCS Reggio Emilia, Reggio Emilia, Italy; 5 Psycho-oncology Unit, Azienda USL-IRCCS Reggio Emilia, Reggio Emilia, Italy

**Keywords:** Palliative care, Communication training, Oncology, Complex intervention

## Abstract

**Background:**

There is widespread agreement about the importance of communication skills training (CST) for healthcare professionals caring for cancer patients. Communication can be effectively learned and improved through specific CST. Existing CSTs have some limitations with regard to transferring the learning to the workplace. The aim of the study is developing, piloting, and preliminarily assessing a CST programme for hospital physicians caring for advanced cancer patients to improve communication competences.

**Methods:**

This is a Phase 0-I study that follows the Medical Research Council framework; this paper describes the following sections: a literature review on CST, the development of the Teach to Talk training programme (TtT), the development of a procedure for assessing the quality of the implementation process and assessing the feasibility of the implementation process, and the pilot programme. The study was performed at a 900-bed public hospital. The programme was implemented by the Specialized Palliative Care Service. The programme was proposed to 19 physicians from 2 departments.

**Results:**

The different components of the training course were identified, and a set of quality indicators was developed. The TtT programme was implemented; all the physicians attended the lesson, videos, and role-playing sessions. Only 25% of the physicians participated in the bedside training. It was more challenging to involve Haematology physicians in the programme.

**Conclusions:**

The programme was completed as established for one of the two departments in which it was piloted. Thus, in spite of the good feedback from the trainees, a re-piloting of a different training program will be developed, considering in particular the bed side component.

The program should be tailored on specific communication attitude and believes, probably different between different specialties.

## Background

There is widespread agreement on the importance of communication skills training (CST) for healthcare professionals caring for cancer patients [1, 2]. The literature indicates that honest and open communication with cancer patients can improve adherence to treatment programmes [3, 4] and lead to benefits for physicians [4–6]. Conversely, poor communication leaves patients alone with their worries and anxiety [7], while professionals become more prone to dissatisfaction and burnout [8].

As highlighted in a number of studies, physician-patient communication can be effectively learned and improved through specific training programmes [2, 9–19]. Nevertheless, the transferability of trainees’ acquired competences to the clinical setting is difficult for range of reasons:

- CST are usually intensive residential programmes held outside trainees’ workplaces and attended by participants from different work environments and setting
- CST are usually implemented by psychologists or experts from psychiatry and behavioural sciences who, de facto, are not directly involved in physician-patient communications
- experiential learning techniques usually employed within these training, such as role playing with peers or trained actors, are not real encounters with real patients, which included in only a minority of programmes.

As many difficult conversations take place in hospitals, all health professionals should be trained to engage in them.

Any hospitals physician should be able to converse about a prognosis, the goals of treatment at the end of life and managing global suffering [20]. To achieve these objectives, a primary palliative care curriculum must be taught, and education about communication issues regarding advanced illness should be the starting point. It is widely documented that the development and implementation of a communication training course is necessary for generalist palliative care physicians to develop core competencies is this area.

Beginning from limitations acknowledged by the literature about existing CST, we developed a novel communication training programme addressed to hospital physicians caring for oncologic patients with palliative care needs, conceived to be implemented inside participants’ workplace (i.e. the hospital), held by specialized palliative care and including encounters with real patients among experiential learning techniques.

The SPIKES protocol, developed in USA with the aim of teaching communication skills at the end of life to medical oncology fellows, was the theoretical model that inspired the programme [21]. It consists in a six steps protocol, each of which is associated with a specific skill (**S**etting up the interview, assessing the patients’ **P**erception, obtaining the patients **I**nvitation, giving **K**nowledge and information to the patients, addressing the patient **E**motion, **S**trategy and **S**ummary).

The training was developed, implemented, assessed and evaluated as a complex intervention [22, 23] according to a phase 0-I of the Medical Research Council (MRC) framework [22, 23].

Our paper describes the phases guiding this process:

- developing a communication training programme focused on hospital physicians caring for advanced cancer patients;
- developing an evaluation system to assess the quality of the implementation;
- launching and preliminarily assessing both the programme and the evaluation system

## Methods

This is a mixed-method, Phase 0-I study that follows the MRC framework for the assessment of complex interventions [22, 23]. According to this framework, it is useful to consider the process of development and evaluation of complex interventions as having several distinct phases. These can be compared with the sequential phases of drug development or may be seen as more iterative. Progression from one phase to another may not be linear. In many cases an iterative process occurs. Preliminary work is often essential to establish the probable active components of the intervention so that they can be delivered effectively during the trial. Identifying which stage of development has been reached in specifying the intervention and outcome measures will give researchers and funding bodies reasonable confidence that an appropriately designed and relevant study is being proposed.

The study was subdivided into three phases.

### Phases of the project

#### Phase 1: developing the communication training programme

SPIKES protocol [21] was the theoretical model that inspired the programme. Besides, we performed a review of systematic reviews on existing communication training programmes with a focus on oncology and palliative care. The PubMed, Embase, Cochrane Library CINHAL and Scopus databases were searched using the MeSH terms [*cancer* OR tumour OR neoplasm OR oncol*] AND [systematic] AND [*communication skills* OR communication strateg* OR *communication training*] for English language articles published until December 2015. An author (S.T.) reviewed the studies’ titles and the abstracts. Screening for full texts was undertaken by two authors (S.D.L. and S.T.).

A sample of physicians who were potentially eligible for the training were preliminarily interviewed, with the aim of gathering information on their perceived training needs in this field and developing the programme accordingly. Interviewed physicians were 4 males and 2 females with a mean age of 52 years (range: 41–67) and an average professional experience of 25 years (range: 18–43). Interviews were analysed qualitatively through thematic analysis [24]. In Table 1 interview’s topic and questions are reported.

**Table 1:** Interview guide on physicians’ perceived training needs

Topic	Question
Training needs	Could you please tell me which are your major difficulties in communicating bad news to advanced cancer patients and their relatives?
	Can you please give me any specific examples?
	According to your opinion which are the major difficulties of your colleagues?
Perceived self-strengths/resources	Regarding your difficulties in bad communication, could you please tell me what are the strengths of your communication, according to your opinion?
Expectations about the training program	Could you please tell me which are your expectations about the training program?

#### Phase 2: assessing the quality of the implementation

This phase was aimed at developing specific procedures to assess the consistency of the implementation process. A set of indicators was developed for a twofold purpose: the first was to assess whether the programme was delivered exactly as outlined and the second was to evaluate each component of the intervention. Thus, information on the objectives achieved or not achieved was collected for each component of the programme (Table 2). The procedure included a semi-structured questionnaire on the perceived usefulness of the programme (Table 3).

**Table 2:** The quality improvement programme with indicators

Dimension	Rationale	Indicators	Expected standard
General training in palliative care	A basic training on palliative care is necessary to educate the future trainees on palliative care topics (e.g., communication)	Proportion of ward physicians attending the 4-h basic training	100%
Request to receive the communication training	A perceived need that training in communication is important for changing future behaviour	Call from the head of the department for communication training	Requested
Developing the documentation for the training	Specific documentation is mandatory	Received the documentation	Received
Didactic lesson	Little basic knowledge on delivering bad news is necessary	Proportion of ward physicians attending the didactic lesson	75% of the participants attend the didactic lesson
Videos	An overview of and a preliminary discussion on different teaching methods prepare students for the didactic lesson	Proportion of ward physicians participating in the video sessions	100%
Role playing	Experiential learning as role playing improve behavioural changes in trainees	Proportion of ward physicians attending at least 2 role playing sessions; proportion of ward physicians performing in at least 2 role playing sessions, at least one as a patient/relative and one as the physician	75%;75%
Bedside trainings	Real-life training improves participants’ awareness of their communication style	Proportion of ward physicians attending at least 3 bed-side sessions	75% of the participants attend the bed-side training
Semi-structured questionnaire on the perceived usefulness of the programme	A self-evaluation of the usefulness of the training components can improve both the structure and contents of the programme	Proportion of physicians attending the whole programme and completing the questionnaires	100%
Bedside training follow up	Follow-up sessions control and re-enforce the maintenance over time of the training course	Proportion of ward physicians performing at least 2 bed-side session follow ups	75%

**Table 3:** Semi-structured questionnaire on the perceived usefulness of the TtT programme

How helpful do you think the 4 components have been with regard to	Delivering bad news to patients and families, % quite/extremely	Exploring patient’s concerns and wishes about illness, % quite/extremely	Building empathy, % quite/extremely
1) Lesson	100	86	86
2) Video screening	100	100	86
3) Role playing	86	100	100
4) Bed-side training	100	100	100

#### Phase 3: assessing feasibility and implementation methods

This phase was aimed at assessing the feasibility of the implementation process within the hospital setting. Both the intervention and the procedure used to assess the quality of the implementation were implemented through a convenience sample of two hospital teams.

We considered the programme feasible if:

- the components of the training course were appropriately identified
- the set of quality indicators was developed and implemented
- the programme was completed as established for the two hospital departments.

Where feasibility was not achieved, the programme included interviews with trainees to detect difficulties and weaknesses of the programme itself (Table 4).

**Table 4:** Interview guide on physicians’ difficulties in completing the programme

Topic	Physicians questions
Project involvement	Could you please tell me what you thought about this training program when you heard about it?
Expectations	What were your expectations in the project?
Perceived benefits/weakness	Regarding this project, could you please tell me what the strengths of this intervention were, according to your opinion? If it impacted on your usual job, how did it do?
	And what about its weakness?
Short report of the experience	Regarding this program, could you please tell me what do you remember about the lesson/the role play?
	Did you complete the training program in all its components?
Future suggestions	As health professional, could you please tell me any suggestions for future training program?

### Population and context

The study was performed at the Arcispedale Santa Maria Nuova of Reggio Emilia. This is a 900-bed Italian research hospital, accredited as a Clinical Cancer Centre by the Organization of European Cancer Institutes (OECI). The Specialized Palliative Care Service (SPCS) is a specialized hospital-based unit with no beds whose mission is to perform clinical, training and research activities in palliative care. The unit was established in 2013, and at present, it includes two senior physicians and three advanced practice nurses, one of whom is devoted to training courses full-time. Psychologists from the hospital Psycho-Oncology Unit cooperate with the SPCS by holding clinical consultations and taking charge of SPCS staff training.

The training was overseen by the two palliative care physicians, the senior nurse specialized in training methodology from the SPCS, and three psychologists from the Psycho-oncology Unit. Based on prior experience in developing and leading communication courses in oncology and palliative care [9, 13, 25, 26], three of the training teachers (S.T., S.D. L and G. A) trained another palliative care physician (S. A.) before the beginning of the programme with the aim of providing her with the competencies needed to act as a teacher.

We proposed the programme to all physicians from the Medical Oncology and Haematology Departments. The Medical Oncology Department provides care for patients with advanced onco-haematological diseases. The department has 20 beds and four physicians. The Haematology Department provides care to haematological patients at all disease stages. The department has 16 beds and 15 physicians. Trainees from both departments were senior physicians. Four were physicians from the Medical Oncology department, four from the Haematology ward, ten from the Haematology day care unit and one from the Haematology home care unit. Only one physician from the Medical Oncology Department had been previously involved in a training course in communication.

The study was approved by the Ethics Committee of Reggio Emilia on 12 June 2015 (n 861/12.6.2015) and was conducted in accordance with the Declaration of Helsinki (http://www.wma.net/e/policy/b3.htm).

### Data analysis

#### Phase I

An author (S.T.) reviewed the studies’ titles and the abstracts for the review of systematic review. Screening for full texts was undertaken by two authors (S.D.L. and S.T.).

The interviews with professionals before the implementation of the programme were recorded, transcribed, and analysed qualitatively with the objective of exploring in detail physicians’ perceived training needs (Table 5). Two researchers (ST and SDL) independently read the transcripts and categorized them into themes [24]. Any disagreement between the researchers was discussed, and a final categorization was determined.

**Table 5:** Themes, sub-themes and representative quotations from qualitative analysis of 6 physicians interviews

Themes and subthemes	Representative quotations
**Communication difficulties**	
Communicating the end of active therapy	“… Trying guide the patients through small steps toward their real situation [the end of curative treatments] is a sort of ‘art of the relationship’, to build through small steps” (Ph 2)
	“When you comes to this point [the end of curative treatments] there is a difficulty in transferring this information to the patient.. This conversation should be anticipated much earlier and not just when you stop the treatment” (ph 3)
Talking about prognosis	“Telling to a patient the prognosis ... There is always something to do but, from that precise moment, you start to lie ... Obviously, I can’t say that there are four weeks of survival left!” (Ph 1)
	“Sometimes there is a sort of omission in communicating a poor prognosis to the hematological patient. This step can really missing …” (Ph 3)
	“Communicating the prognosis to a patient you have known for a long time. We always tend to show the glass half full …” (Ph 4)
Handling interference from relatives	“There are family members who ‘overturn’ the suffering of their loved one not to the disease but the work of health professionals” (ph 1)
	“Situations in which there is an oppositive behavior or even an aggression by family members, and these become the cases that are most difficult to manage” (Ph 2)
	“Families who do not give up, who cannot cut this sort of umbilical cord that unites them with their loved one …” (Ph 3)
	“The relative who continues to search and ask for treatments even when things are over” (Ph 5)
**Source of communication competencies**	
Experience	“I have to say that age and experience help me, so it is easy for me knowing both advanced cancer patient’s previous history and how that history will continue in the future. Therefore, I can also ‘touch’ the sensitive points of what that patient would like to be told, to know …” (Ph 1)
	“It seems to me that I have absorbed some communication techniques ... I would not seem presumptuous” (Ph 2)
	“Our thirty years of experience, in my opinion, is enough!” (Ph 6)
Collaboration with colleagues	“In some situations, your resources are not enough. Then you ask for help to other specialists who will be the psychologist, or the palliative care physician, or your collaborators and colleagues” (Ph 1)
	“I learned communication from briefings, structured meetings, meetings with colleagues on more complex cases” (Ph 3)
	“The confrontation with our team ... with the psychologist” (Ph 4)
	“We improved in keeping a common line when we communicate with patients, and this helps” (Ph 5)
Personal attitude	“Patients and relatives confirm that I can establish a fairly empathic relationship with them. This probably derives from my previous training, from my personality, from my capacity of getting understandably and easily certain speeches” (Ph 2)
	“Surely there is an attitude allowing me to easily establish relationship with patients ... an ability to listen to them ... an attitude in understanding them... adaptability ... sensitivity ...” (Ph 1)
**Expectations toward the training**	
Becoming more empathetic	“Knowing how to leave a little hope even in the face of bad news” (Ph 6)
	“Knowing how to give more consolation when the epilogue cannot be favorable” (Ph 1)
Improving communication with colleagues	“Knowing how to listen more my colleagues, other operators. The clinical eye of the nurse for example” (Ph 3)
	“Improving communication between operators” (Ph 5)
Experiencing less stress	“Approaching myself in a less stressful way in the face of these bad communications that we have to deliver every day” (Ph 4)

#### Phase II

An overview of the objectives achieved and not achieved for each component of the implementation of the programme was obtained through an analysis of the pilot implementation process (Table 2). The answers to the semi-structured questionnaire about the perceived usefulness of each component of the programme (Table 3) were analysed by means of descriptive statistics. The usefulness of each component (i.e. lesson, videoscreening, role playing, bed-side training) was assessed by considering the three main objectives of the training (i.e. delivering bad news, exploring patients’ concerns and supporting them and building empathy).

#### Phase III

The interviews with professionals concerning difficulties encountered by physicians in completing the implementation process (Table 4) were recorded, transcribed, and analysed qualitatively with the objective of exploring in detail reasons related to problems with training completion (Table 6). ST and SDL independently read the transcripts and categorized them into main themes according to the objective of the evaluation. Any disagreement between the researchers was discussed, and a final categorization was determined.

**Table 6:** Themes and representative quotations from qualitative analysis of interviews with physicians who did not complete the training

Themes	Representative quotations
**Global feedback on the training**	*“The only thing was my discomfort facing other people during role play I think that role play should be avoided in the presence of other colleagues. [...] Observer teachers are one thing, because they have to help you see some mistakes, your colleagues are another thing.” (Ph 1)*
	*“It was difficult ... Not so much when you play your part but when we analysed things ... Everyone has difficult cases outstanding ... It was difficult to cope with the return to the memory of cases that I had not yet worked out”. (Ph 4)*
	*“I remember that during the theorethical lesson S.T. [the teacher] stated that we must be able to highlight with the patient the end of his/her life … and that we have to do this in a very clear way … which is something that I do not agree because … at the end you always have to give hope to the patient. Besides, our patients already know when their time has come, so there’s no point in stressing it. Then if we want to underline it with family members, that’s due!” (Ph 1)*
	*“I remember the role plays very well. […]Role plays have impressed me a lot. I don’t remember anything in particular about the theoretical lesson ... “(Ph 3)*
**Organizational issues**	*“Also palliative care physicians have a number of things to do, and this could be a limitation …” (Ph 4)*
	*“With reference to the training, I think that It should be planned having in mind both the characteristics of haematological patients and our great workload” (Ph 2).*
**Misunderstandings about the structure of the programme**	*Interviewer “May I ask you if you have completed all the communication training? Did you carry it out in all its parts?” […]*
	*Ph 1: “Yes, I did. I had one problem only … My distress in front of other persons, during the role-playing sessions …”*
	*“It was a training including either a theoretical, a practical and a field component. I still have to achieve this part because when I needed to communicate some kind of diagnosis, I could not arrange for a meeting with S. [the trainee]. Thus, I still have to do it. I will call S.T. when I will have to communicate a ‘bad’ diagnosis”.(Ph 3)*
**Problems in detecting the “right” situation**	“*I could not attend the bedside sessions because I needed to communicate an illness diagnosis, thus S.T. said that I should call her when I had to perform a truly difficult communication!” (Ph 4)*
	*“Our patients can get worse from one moment to the next, so you make a good plan but ... it’s hard to keep up with this!” (Ph 1)*

## Results

### Phase 1: developing the communication training programme

#### The literature review

**Fig. 1:**
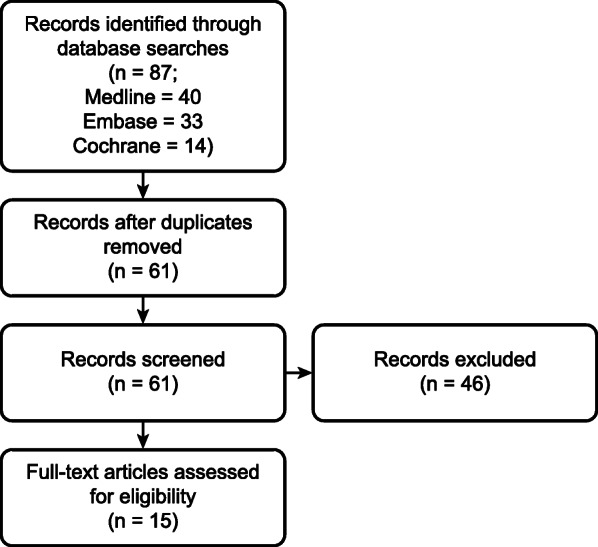
Flow chart

The literature selection process is summarized in the PRISMA diagram (Fig. 1). The search identified 87 records. A total of 61 duplicates were removed, and 43 abstracts were excluded due to ineligibility. Fifteen systematic reviews of CST in oncology and palliative care were included in our review [14, 27–40].

The following recommendations arose from the analysis of the retrieved papers:

- Communication training should be developed and delivered by professionals with both skills and expertise in the field. Facilitators should practice the skills they learn [32, 39, 41].
- Courses should be addressed to small groups of professionals (4–6 persons) [41].
- Successful training courses should last at least 1 day, although there is evidence that the best results come from training courses conducted over a longer period [28, 31, 33, 37, 38].
- Follow-up sessions are also indicated as a promising strategy aimed at reinforcing and maintaining acquired skills over time [17, 38, 41].
- Courses should be learner-centred and practice-oriented and should use a combination of didactic and experiential methods [28, 31, 33–35, 41, 42], such as role playing [17, 28, 34–36, 39, 43].

### The interviews with physicians

A convenient sample of 6 out of 19 physicians participating in the programme were interviewed 1 month before the implementation of the training course to collect their perceived training needs and consequently tailor the contents of the course to them. The major difficulties reported by the trainees concerned three topics: communicating the end of active therapy, talking about prognosis and handling interference from relatives with physicians’ choices with regard to communication with patients about illness. The interviewees considered their communication competencies derived from either field experience, collaboration with colleagues, and nurturing personal attitudes such as sensitivity. Becoming more empathetic in communicating hope, improving communication with colleagues and experiencing less stress and emotional involvement during difficult conversations with patients and their relatives were the interviewees’ expectations regarding the course (Tables 1 and 5).

#### The Teach to Talk programme

According to the literature, existing programmes suffer from a number of limitations: courses are usually residential and are implemented for trainees from different work environments, experiential learning techniques based on role playing with actors make use of simulated scenarios that are very different from real encounters with patients and relatives, facilitators are not directly involved in clinical practice, and training courses are not implemented within the real contexts in which physicians communicate with patients.

Considering both the recommendations and criticisms raised from the literature review as well as the difficulties that emerged from the analysis of the interviews with physicians, we developed a novel intervention named “Teach to Talk” (TtT) training programme. The key features of the programme are the following:

1. the programme is implemented within the participants’ hospital ward, i.e., in the context in which participants are required to practice the communication skills they are learning;
2. the programme includes peer to peer role playing;
3. the programme includes bedside sessions with real patient encounters;
4. teachers are professionals from the hospital SPCS. They are supported by professionals with a psychosocial background, such as psychologists or counsellors.

Inspired by the contents of the SPIKES protocol, the “Teach to Talk” (TtT) programme is aimed at improving physicians’ competencies in the following three broad areas: 1) delivering bad news, 2) exploring patients’ concerns and supporting them, and 3) building empathy.

The SPCS delivers the intervention in five components: video screening, didactic lesson, role playing, bedside training and follow-up. The TtT components as well as the procedures concerning their implementation are described in detail in Table 7.

**Table 7:** The Teach to Talk (TtT) programme

1. **Video screening**. Participants are asked to watch didactic videos representing clinical consultations in which the communication skills involved in the above-mentioned tasks are practiced by actors.	
2. **Didactic lesson**. Subsequently, the participants are provided with an introductory didactic lesson concerning the communication skills needed to deliver bad news, how to explore patients’ concerns and how to build empathy. The discussion between the trainees is focused on both issues from the videos and from the presentation of an actual scenario chosen by the participants. This component is delivered in three hours.	
3. **Role playing.** Beginning in the week after the lesson, role playing sessions are organized, each involving no more than 4 physicians, in which 2 act as actors and the others as observers. Two consecutive role-playing sessions are scheduled at a time, each one lasting about one and half hours. The communication scenarios are proposed by the participants and are based on real situations that they have experienced on the field.	
4. **Bed-side training**. Each participant is supported by a member of the PCT implementing the program in conducting a number of consultations with in- or out-patients, where difficult communication tasks are involved. Bed-side trainings (three per participant) are planned with trainees after the completion of the role play sessions. Each bed-side training is preceded by a briefing aimed at sharing communication objectives and how to manage different scenarios, and the trainings are followed/concluded by a debriefing in which the trainee’s strengths and weaknesses concerning the performed communication tasks are discussed. The *Breaking bad news assessment schedule* (BAS) is used to guide the discussion [44].	
5. **Follow-up.** The follow-up phase takes place 6 months later and consists of 2 bed-side trainings per participant, featuring the structure described above.	
The entire program should be concluded in 6-10 weeks.	

### Phase 2: quality assessment of the programme

The procedure to assess the quality of the programme included a list of indicators covering all of its components (see Table 2). With reference to the *lesson*, *videos, role playing* and *bedside sessions*, a 75% minimum attendance rate was estimated by researchers to be reasonable, which is consistent with the study aims. The time spent by the facilitators teaching the training course was also recorded.

### Phase 3: preliminary assessment of the programme: the evaluation system

The heads of the two departments and all 19 physicians from the two departments agreed to participate in the programme (Table 8). The intervention was implemented between December 2015 and June 2017.These stages are planned as shown in the Gantt Diagram (Table 9).

**Table 8:** Participants demographic characteristics

Department	Sex (male:female)	Age (years) Average	Work experience (years) Average
**Medical Oncology**	2:2	56 (47–67)	27 (12–43)
**Haematology**	10:5	46 (36–60)	16 (4–32)

**Table 9:** The Gantt diagram of the TtT training programme

	Theoretical lesson	Role Play	Bed Side	Follow up bed side
**Medical oncology Department**	November 2015	December 2015	December 2015	December 2015–January 2016
**Hematology Department**	January 2017	February 2017	February-june 2017	Not done

Table 10 summarizes the main findings of the pilot study. The staff from both wards had previously attended general palliative care training. After requesting communication training from the SPCS, these staff members were duly contacted to schedule the preliminary assessment of the participants’ needs for communication training. The issues raised during the interviews were used as a framework to prepare both the lesson and the role-playing sessions. Other topics from the emotional domain were also considered.

**Table 10:** The results of the TtT programme

Dimensions	Medical Oncology Department	Haematology Department
General training in palliative care	100%	100%
Request to receive the communication training	Requested	Requested
Developing the documentation for the training	Received	Received
Didactic lesson	100%	100%
Videos	100%	100%
Role playing	100%	100%
Bedside trainings	100%	6% (1/15)
Semi-structured questionnaire on the perceived usefulness of the programme	100%	20% (3/15)
Bedside training follow up	75% (3/4)	0%

The SPCS prepared the documentation for both the lesson and the role-playing session, as established at the outset. As was laid out in the programme, the training was conducted entirely in the participants’ work environment in small groups. Both the lesson and the role-playing sessions were attended by all the participants. The goals to be achieved during the role playing were changed from those set out in the protocol because the researchers decided to use clinical examples proposed by the trainees. The staff from Medical Oncology completed the entire programme, while those from Haematology completed only part of the programme. Indeed, only one physician completed the entire programme in 6 months. Four did not perform any bedside training sessions. For two of staff members, the trainers did not deem it to be useful for them to complete three bedside training sessions, and the competences they had acquired were judged to be sufficient by the trainers. One physician completed the entire training. The remaining 8 physicians performed only one bed side session.

In addition to the training, follow-up was performed, as established by the programme, by only 3 physicians from the Medical Oncology Department.

The results from the semi-structured questionnaire administered to the physicians who completed the training showed that all the components, particularly the role playing and bedside training sessions, were evaluated as useful or very useful by participants.

Both the didactic lesson and the role-playing sessions were jointly conducted by a palliative care physician from the SPCS and a psychologist from the Psycho-oncology Unit. The synergic approach of the facilitators guaranteed a sort of double perspective in guiding the trainees, both in relation to learning and using appropriate communication skills and to recognizing and managing difficult emotions. Throughout the implementation of the programme, physicians facilitating the bedside training sessions were constantly supervised by the psychologists involved in the project and by a senior nurse training expert. A portfolio was used as a guide to the supervision process.

Findings from the interviews with the four physicians who did not request bedside sessions provide insights into criticisms concerning the implementation of this component, as well as on their comprehensive view on the training. Following, themes emerged from qualitative analysis are briefly described, representative quotations for each theme are reported in Table 6.

#### Global feedback on the training

Two physicians expressed some discomfort in participating to role plays, emphasizing in one case a feeling of embarrassment to be observed by colleagues and in the other an unpleasant sensation of arousal linked to the memory of some emotionally demanding relationships with patients. One physician reported on her disappointment toward a message acknowledged during the theoretical lesson, concerning the relevance of communicating to patients a poor prognosis. On the whole, interviewed physicians highlighted that the educational value of role plays and videos was greater than that of the theoretical lesson.

#### Organizational issues

Some participants referred to practical difficulties in predicting when they have the time to engage themselves in a critical communication with a patient concerning, for example, the end of the active provision of treatment. Problems also emerged because, according to interviewed physicians’ opinion, the trainers themselves were very busy with their clinical activities.

#### Misunderstandings about the structure of the programme

One physician was convinced she had completed the entire training course, while another was still trying to arrange an encounter with the trainer.

#### Problems in detecting the “right” situation

A physician explained that, during the training, she had to communicate bad news concerning only illness diagnosis, a task perceived as less challenging and difficult than communicating a poor prognosis. Another physician highlighted her difficulty in knowing in advance whether she should have to cope with a difficult communication scenario due to rapid changes in patients’ condition.

## Discussion

This study focused on the development of a communication training programme, indicators of the fidelity of the implementation, the different components of the intervention and its preliminary assessment. The programme was completed as established for one of the two departments in which it was piloted; for the Haematology department, bedside training and the consequent follow-up sessions were missing. Thus, in spite of the high perceived utility expressed from the trainees, major changes are needed to ensure the feasibility of this training program.

We developed our intervention and included all the components evaluated as essential in the last ASCO statements [45]. We chose to offer only one lesson, which was attended by all the participants from both wards, and we used role-playing between peers to allow for safe interaction between colleagues within the small-group setting and peer-reviewed the feedback, which a number of studies stated were effective tools [36, 46–54]. This approach was also highlighted in our pilot study, where participants evaluated the role playing sessions in which they took part as highly useful.

Regarding the bedside training, recent studies [55, 56] have suggested and proved the importance of coaching after didactic modules because of its focus on individual learning goals and the possibility of tailoring training to personal weaknesses. One-to-one coaching by palliative care physicians was also the main tool used in the study by Clayton et al. [56] on a group of voluntary, junior doctors. Satisfaction with the course was expressed by the participants, but only one-third of the participants saw improvement in their communication skills.

In our pilot study, most physicians from the Haematology ward did not receive this coaching session (bedside training), even though there had been a formal request by the heads of the ward to participate in the training and the training met the specific needs expressed by physicians during the preliminary need assessment.

Two main reasons could explain the major limitation of our training programme: first, haematologists must address organizational issues, as declared in some interviews; second, theoretical and cultural issues underlying the haematologists’ concept of palliative care and the palliative care approach should be taken into account as contributory factors.

The *Teach to Talk* programme has been implemented since 2015 by an SPCS inside the hospital. The interaction with the Haematology ward is well documented by the increasing number of year-to-year consultation requests. Interaction between palliative care and haematology has been explored by recent literature. Although the value of palliative care is recognized by haematologists, there still seems to be resistance to the reality and practicalities associated with the referral of haematologic patients to palliative care services [57].

In literature a great amount of evidence underscores the difficult of hematologists to recognize patients’ poor prognosis and talk with them about it [58–60]: in the study by Alexander, a lack of patients involvement in decision about treatment, as well a tendency to avoid prognostic discussion emerge in the analysis of video recorded real encounters with patients. Hematologists participants in a qualitative research [57] acknowledge taking a paternalistic approach towards certain decisions and explained their therapeutic optimism in order to bolster patients in toxic but curative treatments. The intention ‘*not to give up’* was strengthened by the intense physician-patient relationship and by the unpredictable nature of the treatment itself [57]. The hematologic patient is described differently from the oncologic one for the no predictable disease’s trajectory [60, 61] thus, the right moment to share a bad communication could be not so clear.

These difficulties were similar with problems raised by the haematologists in our study, for instance, with regard to the appropriate time to communicate with patients regarding the turning point of an illness (e.g., the end of active treatment or disease leading to poor prognosis). An international trial by Szekendi et al. [62] highlighted the impact of embedding a palliative care team with a selected non-palliative care service: non-palliative care physicians report an increase in comfort as well as in their skills in conducting care conversations.

As far as we know, few training courses in communication are addressed to haematologists and thus focus on their specific communication needs [63]. At the same time, communication remains a challenge for haematologists. Formal communication skills training and target interventions for patients with haematologic malignancies by palliative care staff have been called for by some authors [64, 65].

We developed a set of indicators to assess the quality of the implementation. We propose to take these indicators into account in every setting to expedite implementation. In particular, we believe the preliminary stages (general training in palliative care, requests to receive the communication training, communication need assessment) to be mandatory to improve core competencies in basic palliative care for other professionals.

Findings from this study need to be interpreted by acknowledging some limitations. We have initiated the programme at only one clinical cancer centre. Nevertheless, we launched our project within a coherent methodological framework. This approach involved the recommendation to assess the local feasibility of complex interventions so the project can be amended as necessary and evaluated on a larger scale.

The results of this study strongly suggest the need for developing a revised version of the TtT programme. In hospital settings, the duration of the intervention should be longer than 8 weeks, depending on the specific characteristics of the ward in which it is implemented (e.g., number of physicians, professionals’ training needs, frequency of bad news communication, organization of work). The number of bedside sessions per participant should be determined on the basis of the competencies acquired by single participants throughout the training in accordance with the facilitator’s judgement.

Bedside sessions should be scheduled a priori with facilitators rather than self-managed by participants because self-management, an active and proactive behaviour, may facilitate concrete change in communication attitudes.

## Conclusions

In the last 10 years, research from the literature emphasized that training in communication skills is not enough to bring about real change in professional attitudes [19, 27, 66, 67]. We implemented an educational intervention with a well-integrated palliative care team in order to overcome limitations of existing residential training programmes and to impact communicative behaviour in the contexts where professionals actually work. However, major changes are needed to ensure the feasibility of this training programme.

Turrillas et al. [68] argue that the most effective training method should be tailored to the environment and context. A re-piloting of a different training program will be developed, considering in particular the bed side component.

Moreover the program should be tailored on specific communication attitude and believes, probably different between different specialties as emerged in our interviews to haematologists.

## Data Availability

The study documentation is collected and managed by the coordinator of the study centre (PC Unit, AUSL – IRCCS di Reggio Emilia), and datasets are available on reasonable request.

## Abbreviations

MRC: Medical Research Council
SPCS: Specialized palliative care service
CST: Communication skill training
TtT: Teach to talk
ASCO: American society of clinical oncology
BAS: Breaking bad news assessment schedule
